# Cord blood levels of insulin‐like growth factor‐1 and insulin‐like growth factor binding protein‐3 correlate with perinatal brain development in fetal congenital heart disease

**DOI:** 10.1002/uog.29271

**Published:** 2025-06-28

**Authors:** M. Nijman, N. H. P. Claessens, J. M. P. J. Breur, L. Reijn, N. J. G. Jansen, I. M. van Ooijen, R. de Heus, C. H. Nijboer, M. N. Bekker, M. J. N. L. Benders, R. Stegeman, J. E. G. Vaes, the CHD LifeSpan Study Group Utrecht, Joppe Nijman, Thomas Alderliesten, Rian Bosch, Roelie M. Wösten‐van Asperen, Henriette ter Heide, Trinette J. Steenhuis, Martijn G. Slieker, Gabrielle G. van Iperen, Kim van Loon, Boyd V. Martherus, Erik Koomen, Abraham van Wijk, Paul H. Schoof, Hanna Talacua, Neeltje Crombag, Bauke M. Adriaanse, Niels Blanken, Rutger A.J. Nievelstein, Monique M.J. van Schooneveld, Lotte Soeters, Maaike C.A. Sprong

**Affiliations:** ^1^ Department of Neonatology Wilhelmina Children's Hospital, University Medical Center Utrecht Utrecht The Netherlands; ^2^ Department of Pediatric Cardiology Wilhelmina Children's Hospital, University Medical Center Utrecht Utrecht The Netherlands; ^3^ Department of Pediatrics Wilhelmina Children's Hospital, University Medical Center Utrecht Utrecht The Netherlands; ^4^ Department of Pediatrics Beatrix Children's Hospital, University Medical Center Groningen Groningen The Netherlands; ^5^ Center for Image Sciences High Field MR Research, University Medical Center Utrecht Utrecht The Netherlands; ^6^ Department of Obstetrics and Gynecology St Antonius Hospital Utrecht The Netherlands; ^7^ Department of Obstetrics and Gynecology University Medical Center Utrecht Utrecht The Netherlands; ^8^ Department for Developmental Origins of Disease Wilhelmina Children's Hospital, University Medical Center Utrecht Utrecht The Netherlands; ^9^ Department of Pediatrics Willem Alexander Children's Hospital, Leiden University Medical Center Leiden The Netherlands

**Keywords:** brain development, brain volume, congenital heart disease, fetus, IGF‐1, IGFBP‐3, magnetic resonance imaging, neonate, trophic factors

## Abstract

**Objectives:**

Neonates with critical congenital heart disease (CCHD) are at risk for adverse early brain development and long‐term neurodevelopmental sequelae. Insulin‐like growth factor‐1 (IGF‐1) and insulin‐like growth factor binding protein‐3 (IGFBP‐3) are essential contributors to brain growth and maturation. The present study aimed to compare cord blood levels of IGF‐1 and IGFBP‐3 between neonates with CCHD and healthy controls, and to explore their association with perinatal brain development.

**Methods:**

This was a prospective, observational study conducted at Wilhelmina Children's Hospital, Utrecht, The Netherlands, between June 2019 and March 2022. The study cohort comprised term neonates diagnosed prenatally with a severe cardiac defect on ultrasound, for which surgical repair within the first month after birth was anticipated. IGF‐1 and IGFBP‐3 levels were measured from cord blood samples obtained directly after birth in term neonates with CCHD and healthy control neonates. All cases were stratified into two subgroups according to the predicted cerebral oxygen delivery (COD) *in utero* (i.e. normal or lower), based on the expected impact of the cardiac diagnosis on COD. In cases with CCHD, fetal and preoperative neonatal brain magnetic resonance imaging (MRI) scans were performed. Regional and total brain volumes were assessed, after adjustment for sex and postmenstrual age at MRI, using linear regression equations.

**Results:**

Cord blood samples were collected from 39 neonates with CCHD and 20 healthy controls. IGFBP‐3 levels were significantly lower in cases of CCHD *vs* healthy controls (median, 0.89 mg/L *vs* 1.09 mg/L; *P* = 0.027). When comparing the two subgroups of predicted COD, cases with lower COD (*n* = 30) displayed reduced IGF‐1 (median, 9.7 nmol/L *vs* 11.7 nmol/L; *P* = 0.003) and IGFBP‐3 (median, 0.88 mg/L *vs* 1.07 mg/L; *P* = 0.005) levels compared to those with normal COD (*n* = 29). IGF‐1 levels were associated positively with neonatal cortical gray matter (*R* ^2^, 0.13), deep gray matter (*R* ^2^, 0.15) and total brain (*R* ^2^, 0.10) volumes (all *P* < 0.05). Positive associations were identified between IGFBP‐3 and neonatal cortical gray matter (*R* ^2^, 0.07) as well as cerebellar (*R* ^2^, 0.11) volumes (both *P* < 0.05). Furthermore, IGF‐1 was correlated positively (*r* = 0.47; *P* = 0.039) with fetal‐to‐neonatal cortical gray matter growth per week.

**Conclusions:**

IGFBP‐3 levels were demonstrated to be reduced in neonates with CCHD, and cases with lower predicted COD demonstrated decreased levels of IGF‐1 and IGFBP‐3. In addition, lower IGF‐1 and IGFBP‐3 levels at birth were associated with reduced early volumetric brain development. These results indicate that a compromised oxygenation profile *in utero* in fetuses with CCHD may influence the IGF axis, possibly contributing to impaired brain growth in CCHD. © 2025 The Author(s). *Ultrasound in Obstetrics & Gynecology* published by John Wiley & Sons Ltd on behalf of International Society of Ultrasound in Obstetrics and Gynecology.

## Introduction

Congenital heart disease (CHD) is the most prevalent anomaly at birth, affecting approximately 1% of newborns^1^. Beyond the impact on cardiovascular health, children with CHD are at risk of lifelong neurodevelopmental sequelae, such as behavioral, cognitive, executive functioning and motor impairments. These neurological abnormalities stand out as the most frequent extracardiac comorbidities of CHD and may affect quality of life^2^, ^3^.

The complex interplay between CHD and compromised neurodevelopment begins during the prenatal stage. Multiple fetal imaging studies have shown that fetuses with CHD display smaller brain volume, reduced cortical folding, white matter immaturity and abnormal cerebral metabolism^4^, ^5^, ^6^, ^7^, ^8^, ^9^. The etiology for abnormal fetal brain development is thought to be multifactorial, including reduced cerebral substrate delivery due to the nature of the cardiac defect, genetic factors and an adverse intrauterine environment^4^, ^10^, ^11^, ^12^. However, further exploration of the biological mechanisms underlying impaired *in‐utero* brain development is important to establish prenatal targets for neuroprotection in cases of CHD.

An essential endocrine regulator of fetal organogenesis is insulin‐like growth factor‐1 (IGF‐1)^13^. IGF‐1 is synthesized by all fetal tissues and promotes cell proliferation, survival and differentiation. In the fetal brain, IGF‐1 receptors are expressed from early in gestation, exerting distinct trophic effects on neurodevelopmental processes, such as synaptogenesis, plasticity and myelination^14^, ^15^. Insulin‐like growth factor binding protein‐3 (IGFBP‐3) serves as the primary carrier protein for IGF‐1, especially in the brain, and regulates IGF‐1 activity by delivery of this hormone to cell receptors, resulting in the activation of signaling pathways^16^.

Prior clinical research has implied that hypoxia and inflammation lead to downregulation of IGF‐1 and IGFBP‐3^17^, ^18^, ^19^, ^20^, ^21^, ^22^, which has been associated with impaired somatic growth in CHD as well as reduced neonatal brain volume and poorer neurodevelopmental outcomes in preterm infants^23^, ^24^, ^25^, ^26^, ^27^, ^28^. IGF‐1 and IGFBP‐3 levels and their interplay with early brain development have not yet been studied in neonates with CHD. This study aimed to address this gap by prospectively comparing IGF‐1 and IGFBP‐3 levels in cord blood between neonates with critical CHD (CCHD) and healthy controls, as well as between cases with normal *vs* lower predicted cerebral oxygen delivery (COD) *in utero*. In addition, in neonates with CCHD, we explored the association between IGF‐1 and IGFBP‐3 levels and total and regional volumetric brain growth, using fetal and neonatal magnetic resonance imaging (MRI).

## Methods

### Study population

This prospective, observational study was conducted at Wilhelmina Children's Hospital, Utrecht, The Netherlands, between June 2019 and March 2022. The CCHD cohort consisted of term neonates diagnosed prenatally with a severe cardiac defect on ultrasound for which surgical repair within the first month after birth was anticipated. Exclusion criteria for the CCHD cohort included confirmed or suspected genetic disorder, multiple gestation, major extracardiac anomaly and maternal diabetic disorder. Cases with CCHD were selected sequentially from a larger prospective study (CHD LifeSpan Study, MREC no. 16‐093). For the present substudy, umbilical cord blood samples were collected from 50 neonates. For the control cohort, 20 term neonates delivered by elective Cesarean section following an uncomplicated singleton pregnancy were selected sequentially for inclusion. The clinical indication for these elective Cesarean sections was either breech position of the fetus or maternal history of a previous Cesarean section.

Ethical approval for both the larger prospective study and present substudy was granted by the local Institutional Review Board (MREC no. 16‐093 and 19‐071, respectively). Parental written informed consent for the use of clinical data for research purposes was acquired for the CCHD cohort. The Institutional Review Board waived the requirement for parental written informed consent for the control cohort.

### IGF‐1 and IGFBP‐3 measurement

Blood samples were collected from the umbilical vein directly after delivery, following clamping of the umbilical cord. The blood samples underwent centrifugation and the resulting serum was stored at −20°C. IGF‐1 levels were measured by chemiluminescence immunoassay using a Liaison XL analyzer (Diasorin, Saluggia, Italy) with a detection range of 1.3–195 nmol/L and intra‐assay precision of 4.9–7.1%. IGFBP‐3 levels were quantified using radioimmunoassay (Wallac Wizard Gamma Counter; PerkinElmer, Waltham, MA, USA) with a lower detection limit of 0.002 mg/L and intra‐assay precision of 4.2–5.8%. To decrease interassay variability, harmonization samples from the IGF‐1 harmonization program in The Netherlands (SD‐NL) were used.

### Classification of fetal cerebral oxygen delivery

Since prior studies indicated that a hypoxic state negatively impacts IGF‐1 and IGFBP‐3 levels^17^, ^18^, ^21^, ^22^, we created two subgroups within our study cohort based on the predicted impact of the cardiac diagnosis on fetal COD. The first subgroup, designated as the lower COD group, comprised cases in which the fetal COD was predicted to be mildly to severely reduced (e.g. aortic arch anomalies with a large ventricular septal defect, hypoplastic left heart syndrome and transposition of the great arteries). The second subgroup, referred to as the normal COD group, included the control cohort and cases with cardiac diagnoses in which the fetal COD was expected to be within the normal range (e.g. aortic arch anomalies with or without a small ventricular septal defect). This classification system was adapted from Rollins *et al*.^29^ and Cromb *et al*.^30^. A pediatric cardiologist (J.M.P.J.B) reviewed all cases and assigned them to one of the two COD subgroups based on fetal echocardiography reports.

### Fetal and neonatal MRI acquisition

Cases with CCHD were managed according to a standardized clinical protocol that included fetal MRI in the third trimester and preoperative neonatal MRI, performed on a 3.0 Tesla MRI system (Philips Medical Systems, Best, The Netherlands). The fetal scanning protocol included T2‐weighted MRI in the axial, sagittal and coronal planes, with an echo time of 180 ms, slice thickness of 2.5 mm, acquisition voxel size of 1.25 × 1.25 mm, reconstructed voxel size of 0.70 × 0.70 mm and field‐of‐view of 360 × 360 × 101 mm. The neonatal scanning protocol included coronal T2‐weighted MRI (echo time of 150 ms, slice thickness of 1.2 mm, acquisition voxel size of 0.78 × 0.78 mm, reconstructed voxel size of 0.35 × 0.35 mm and field‐of‐view of 180 × 180 × 132 mm), coronal three‐dimensional (3D) T1‐weighted MRI, axial diffusion‐weighted imaging, axial susceptibility‐weighted imaging and 3D MRI venography. The complete fetal and neonatal scanning protocols and procedures have been published previously^5^. No imaging was performed in the control cohort.

All images were analyzed jointly by two reviewers (N.H.P.C., M.N.) for the presence of white matter injury, arterial ischemic stroke, hypoxic‐ischemic watershed injury, hemorrhage (intraparenchymal, intraventricular, subdural, cerebellar) and sinovenous thrombosis, using the standardized scoring protocol described by Stegeman *et al*.^31^. During the scoring of brain injury and the assessment of image quality, the reviewers were blinded to IGF‐1 and IGFBP‐3 levels to minimize potential bias.

### Fetal imaging processing

We applied the automated 3D slice‐to‐volume registration reconstruction method to the fetal MRI scans, resulting in reconstruction and motion correction of the images (https://github.com/SVRTK/auto‐proc‐svrtk)^32^, ^33^. The resulting reconstructions were upsampled to 0.5 mm^3^ isotropic resolution and segmented using the automated brain volumetry and automated parcellation for 3D fetal MRI (BOUNTI) pipeline^34^. We reviewed the quality of all reconstructions and segmentations and excluded cases with poor quality. Poor quality was defined as having > 10% of slices affected by motion or segmentation errors. Total brain volume was computed as the sum of the cortical gray matter, white matter, deep gray matter, cerebellum and brainstem.

### Neonatal imaging processing

To quantify total and regional neonatal brain volumes, the structural processing pipeline of the developing Human Connectome Project (dHCP) was used^35^. With this approach, the coronal T2‐weighted MRI data underwent reconstruction, bias correction and brain extraction. Following these steps, volumetric tissue segmentation was carried out using an automated segmentation algorithm for the neonatal brain^36^. We visually inspected all segmented images for quality assurance: minor segmentation errors were corrected with ITK‐SNAP 3.6.0 (www.itksnap.org)^37^, but images with notable motion artifacts or segmentation errors in > 10% of slices were excluded from the analysis. Total brain volume was calculated as the sum of all tissue labels (i.e. type of brain tissue), excluding the ventricles and extracerebral fluid. For analysis of regional brain volumes, we included the tissue labels representing cortical gray matter, white matter, deep gray matter and cerebellum.

### Statistical analysis

The statistical analyses were performed using SPSS version 29.0 (IBM Corp., Armonk, NY, USA). Normality of data was assessed using a combination of histograms, Q–Q plots, the Kolmogorov–Smirnov test and the Shapiro–Wilk test. Descriptive statistics were applied to summarize baseline characteristics, with categorical data presented as *n* (%) and continuous data presented as mean ± SD or median (interquartile range (IQR)). Pearson's chi‐square test, Fisher's exact test, independent samples *t*‐test and Mann–Whitney *U*‐test were used to assess differences in baseline characteristics between the control cohort and CCHD cohort, and between cases with normal predicted COD and those with lower predicted COD. The Mann–Whitney *U*‐test was used to compare cord blood levels of IGF‐1 and IGFBP‐3 between the control and CCHD cohorts, as well as between cases with normal predicted COD and those with lower predicted COD.

Neonatal brain volumes were adjusted for sex and postmenstrual age at MRI using linear regression equations, following the methodology outlined by Sadhwani *et al*.^9^; however, in contrast to that fetal imaging study, we corrected neonatal brain volumes for linear postmenstrual age at MRI rather than for quadratic postmenstrual age at MRI, because we observed a more linear relationship between neonatal brain volumes and postmenstrual age at MRI. As described in the study of Sadhwani *et al*., the residual brain volume indicates the disparity between the observed brain volume and the expected value for each neonate^9^. The residual brain volumes were calculated for each brain tissue label within the CCHD cohort using linear regression models that included absolute brain volume (dependent variable), postmenstrual age at MRI (independent variable) and neonatal sex (independent variable).

Linear regression analyses were used to assess the association between neonatal residual total brain volume and regional brain volume (dependent variable) and IGF‐1 and IGFBP‐3 (independent variables), while controlling for birth mode (vaginal *vs* Cesarean delivery). Because of the size of our cohort, we did not perform separate linear regression analyses for the lower COD subgroup and the normal COD subgroup.

Fetal‐to‐neonatal growth rates of the cortical gray matter, white matter, cerebellum and total brain volume per week were computed for each individual by subtracting the fetal brain volumes from the neonatal brain volumes and dividing the result by the time interval between the two scans. Partial correlational analysis was employed to evaluate the relationship of IGF‐1 and IGFBP‐3 with the fetal‐to‐neonatal growth rate per week in the CCHD cohort, controlling for fetal sex and birth mode.

Twenty‐seven (69%) of the neonates in the CCHD cohort are also participants in an ongoing, double‐blinded, postnatal neuroprotective trial (NCT0421 7421)^38^; therefore, we did not perform an analysis of the association between IGF‐1 or IGFBP‐3 and acquired brain injury.

As this was a pilot study, no formal sample size calculations were performed. In addition, given the explorative nature of the study, a two‐tailed significance level of *P* < 0.05 was applied.

## Results

### Clinical characteristics

**Figure 1:**
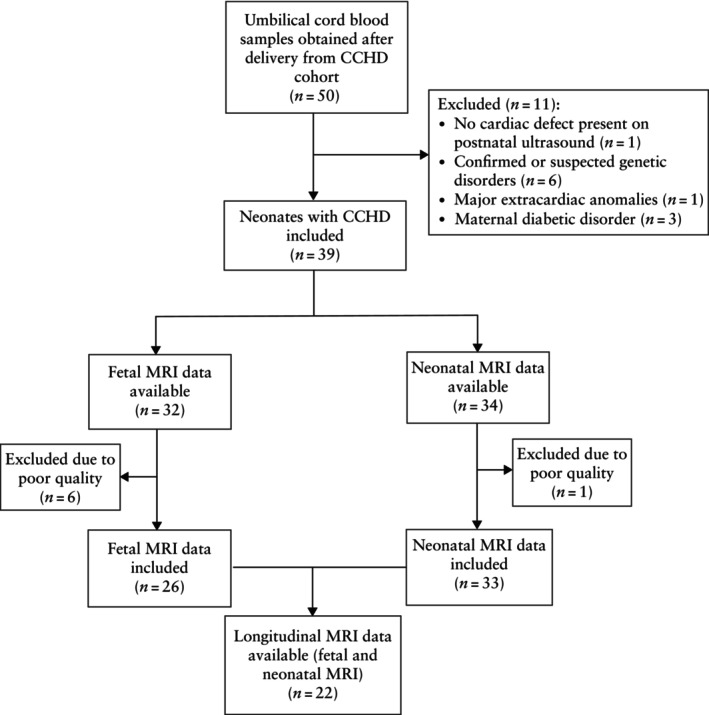
Flowchart showing inclusion of patients with critical congenital heart disease (CCHD) and included fetal and neonatal magnetic resonance imaging (MRI) scans from this cohort.

A total of 39 neonates with CCHD and 20 control neonates were included in the study (Figure 1). There were nine neonates with normal predicted COD and 30 with lower predicted COD in the CCHD group, resulting in a total of 29 cases with normal predicted COD and 30 cases with lower predicted COD. The demographic and clinical characteristics of the study cohort are outlined in Table 1. The CCHD group had a significantly higher proportion of male neonates than the control cohort (79.5% *vs* 45.0%; *P* = 0.007), as well as slightly lower 5‐min Apgar scores (median, 9.0 (IQR, 9.0–10.0) *vs* 10.0 (IQR, 10.0–10.0); *P* < 0.001). All control cases were delivered via Cesarean section, compared with only three (7.7%) cases in the CCHD cohort, representing a significant difference in delivery method (*P* < 0.001).

**Table 1:** Clinical characteristics of the study cohort, which included neonates with critical congential heart disease (CCHD) and healthy controls, stratified according to their expected cerebral oxygen delivery (COD) *in utero*

Characteristic	Control cohort (*n* = 20)	CCHD cohort (*n* = 39)	*P*	Normal COD (*n* = 29)	Lower COD (*n* = 30)	*P*
Maternal						
Gravidity	2.0 (2.0–3.0)	2.0 (2.0–3.0)	0.406	2.0 (2.0–3.0)	2.0 (1.0–3.0)	0.912
Parity	1.0 (0.3–1.0)	1.0 (0.0–2.0)	0.959	1.0 (1.0–1.5)	1.0 (0.0–2.0)	0.431
Smoking	1 (5.0)	0 (0)	0.339	1 (3.4)	0 (0)	0.492
Pregnancy complications	0 (0)	2 (5.1)	0.544	0 (0)	2 (6.7)	0.492
Gestational hypertension	0 (0)	1 (2.6)		0 (0)	1 (3.3)	
Pre‐eclampsia	0 (0)	1 (2.6)		0 (0)	1 (3.3)	
Neonatal						
Male sex	9 (45.0)	31 (79.5)	0.007	15 (51.7)	25 (83.3)	0.009
Gestational age at birth (weeks)	39.0 (38.7–39.4)	39.0 (38.7–39.3)	0.590	39.0 (38.7–39.4)	39.0 (38.7–39.3)	0.548
Birth weight (g)	3382.3 ± 350.3	3249.44 ± 434.9	0.242	3454.1 ± 415.1	3140.2 ± 346.2	0.003
Birth‐weight *Z*‐score	0.04 ± 0.8	−0.33 ± 1.1	0.193	0.21 ± 1.00	−0.62 ± 0.88	0.002
5‐min Apgar score	10.0 (10.0–10.0)*	9.0 (9.0–10.0)†	<0.001	10.0 (9.5–10.0)	9.0 (9.0–10.0)†	<0.001
Mode of delivery			<0.001			<0.001
Spontaneous vaginal delivery	0 (0)	4 (10.3)		1 (3.4)	3 (10.0)	
Induced vaginal delivery	0 (0)	32 (82.1)		7 (24.1)	25 (83.3)	
Elective CS	20 (100)	2 (5.1)		21 (72.4)	1 (3.3)	
Secondary CS	0 (0)	1 (2.6)		0 (0)	1 (3.3)	

Data are given as median (interquartile range), *n* (%) or mean ± SD. *P* < 0.05 indicates statistical significance.

* Data missing for four cases.

† Data missing for one case. CS, Cesarean section.

Cases with lower predicted COD had a significantly lower mean birth weight than those with normal predicted COD (3140.2 ± 346.2 *vs* 3454.1 ± 415.1 g; *P* = 0.003) and mean birth‐weight *Z*‐score (–0.62 ± 0.88 *vs* 0.21 ± 1.00; *P* = 0.002).

The types of cardiac defects diagnosed on prenatal ultrasound are presented in Table 2 and the types of preoperative brain injury detected on neonatal MRI are given in Table 3.

**Table 2:** Type of cardiac defect diagnosed on prenatal ultrasound in neonates with critical congenital heart disease (CCHD), overall and according to their expected cerebral oxygen delivery (COD) *in utero*

Cardiac defect	CCHD cohort (*n* = 39)	Normal COD (*n* = 9)	Lower COD (*n* = 30)
Transposition of great arteries	15 (38.5)	0 (0)	15 (50.0)
Hypoplastic left heart syndrome	4 (10.3)	0 (0)	4 (13.3)
Hypoplastic right heart syndrome	1 (2.6)	0 (0)	1 (3.3)
Complex single ventricle with pulmonary obstruction	2 (5.1)	0 (0)	2 (6.7)
Aortic coarctation and arch hypoplasia with large VSD	2 (5.1)	0 (0)	2 (6.7)
Aortic coarctation and arch hypoplasia without or with small VSD	1 (2.6)	1 (11.1)	0 (0)
Aortic coarctation with small VSD	1 (2.6)	1 (11.1)	0 (0)
Borderline left heart with large VSD	2 (5.1)	0 (0)	2 (6.7)
Borderline left heart without or with small VSD	7 (17.9)	7 (77.8)	0 (0)
Interrupted aortic arch with large VSD	1 (2.6)	0 (0)	1 (3.3)
Tetralogy of Fallot	1 (2.6)	0 (0)	1 (3.3)
Truncus arteriosus	1 (2.6)	0 (0)	1 (3.3)
Pulmonary atresia	1 (2.6)	0 (0)	1 (3.3)

Data are given as *n* (%). VSD, ventricular septal defect.

### IGF‐1 and IGFBP‐3 levels in cord blood

**Figure 2:**
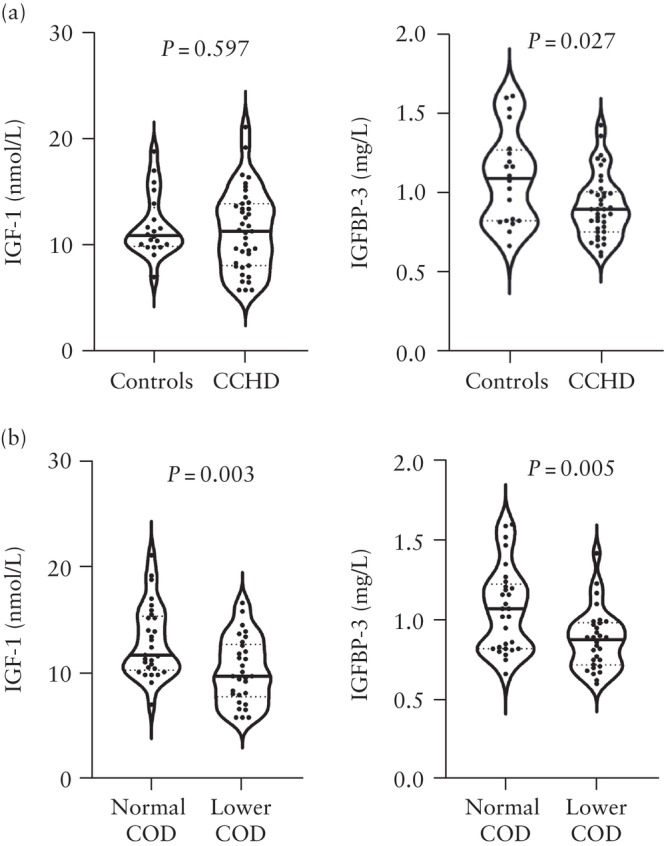
Distribution of cord blood insulin‐like growth factor‐1 (IGF‐1) and insulin‐like growth factor binding protein‐3 (IGFBP‐3) levels in: (a) the control cohort *vs* cohort with critical congenital heart disease (CCHD); and (b) between cases with predicted normal cerebral oxygen delivery (COD) *vs* lower COD. The Mann–Whitney *U*‐test was used for comparisons and congenital heart disease cohort, as well as between cases with predicted normal or lower. *P* < 0.05 indicates statistical significance.

Figure 2 displays the distribution of the cord blood levels of IGF‐1 and IGFBP‐3 in the control cohort, CCHD cohort and the two COD subgroups. No notable difference was found in IGF‐1 levels between controls and neonates with CCHD (median, 10.9 (IQR, 9.9–13.5) nmol/L *vs* 11.3 (IQR, 8.1–13.9) nmol/L; *P* = 0.597) (Figure 2a). However, when comparing the two COD subgroups, significantly lower levels of IGF‐1 in cases with lower COD were observed (median, 9.7 (IQR, 7.7–12.7) nmol/L *vs* 11.7 (IQR, 10.3–15.4) nmol/L; *P* = 0.003) (Figure 2b).

Furthermore, the median concentration of IGFBP‐3 was lower in the CCHD cohort than in the control cohort (0.89 (IQR, 0.75–1.00) mg/L *vs* 1.09 (IQR, 0.82–1.26) mg/L; *P* = 0.027) (Figure 2a). Within the COD subgroups, cases with lower COD had reduced IGFBP‐3 levels compared to those with normal COD (median, 0.88 (IQR, 0.72–0.98) mg/L *vs* 1.07 (IQR, 0.82–1.23) mg/L; *P* = 0.005) (Figure 2b).

IGF‐1 levels showed a significant association with birthweight *Z*‐score in the total study cohort (*β* coefficient, 0.16 (95% CI, 0.10–0.23); adjusted *R* ^2^, 0.29; *P* < 0.001), while this association was not evident for IGFBP‐3 (*β* coefficient, 0.99 (95% CI, −0.09 to 2.06); adjusted *R* ^2^, 0.04; *P* = 0.070). In our study population, neither IGF‐1 nor IGFBP‐3 levels were associated with gestational age at birth.

**Table 3:** Type of preoperative brain injury diagnosed on neonatal magnetic resonance imaging (MRI) in cases with critical congenital heart disease (CCHD), overall and according to their expected cerebral oxygen delivery (COD) *in utero*

Brain injury	CCHD cohort (*n* = 33)*	Normal COD (*n* = 8)	Lower COD (*n* = 25)
White matter injury	9 (27.3)	1 (12.5)	8 (32.0)
Stroke	2 (6.1)	0 (0)	2 (8.0)
Hypoxic‐ischemic watershed injury	0 (0)	0 (0)	0 (0)
Subdural hemorrhage	14 (42.4)	1 (12.5)	13 (52.0)
Intraventricular hemorrhage	5 (15.2)	1 (12.5)	4 (16.0)
Intraparenchymal hemorrhage	0 (0)	0 (0)	0 (0)
Cerebellar hemorrhage	3 (9.1)	1 (12.5)	2 (8.0)
Sinovenous thrombosis	0 (0)	0 (0)	0 (0)

Data are given as *n* (%).

* Data on brain injury findings on preoperative neonatal MRI were available for 33 cases of CCHD.

### Neonatal brain volume and IGF‐1/IGFBP‐3 levels

Within the CCHD cohort, 33 (85%) neonates had a postnatal preoperative cerebral MRI scan available with sufficient quality for volumetric analyses (Figure 1). The included scans were performed at a median postnatal age of 3.0 (IQR, 2.0–5.0) days and median postmenstrual age of 39.7 (IQR, 39.0–39.9) weeks. Brain injury was identified in 22 (66.7%) neonates, with subdural hemorrhage (42%), white matter injury (27%) and intraventricular hemorrhage (15%) being the most prevalent lesions (Table 3).

Significant positive associations were noted between IGF‐1 and residual volumes of the cortical gray matter (*β* coefficient, 1.46 (95% CI, 0.31–2.61); adjusted *R* ^2^, 0.13; *P* = 0.015), deep gray matter (*β* coefficient, 0.20 (95% CI, 0.05–0.34); adjusted *R* ^2^, 0.15; *P* = 0.009) and total brain (*β* coefficient, 2.62 (95% CI, 0.31–4.93); adjusted *R* ^2^, 0.10; *P* = 0.028). IGFBP‐3 demonstrated significant positive associations with the residual volumes of the cortical gray matter (*β* coefficient, 23.46 (95% CI, 0.44–46.47); adjusted *R* ^2^, 0.07; *P* = 0.046) and cerebellum (*β* coefficient, 4.12 (95% CI, 0.62–7.62); adjusted *R* ^2^, 0.11; *P* = 0.023) (Table 4).

**Table 4:** Linear regression models for assessment of associations between neonatal brain volumes and insulin‐like growth factor‐1 (IGF‐1) and insulin‐like growth factor binding protein‐3 (IGFBP‐3)

Tissue label	Mean ± SD	β coefficient (95% CI)	*P*	Adjusted *R* ^2^
IGF‐1 and brain volume				
Residual total brain volume (mL)	0.00 ± 26.36			0.10
IGF‐1 (nmol/L)		2.62 (0.31 to 4.93)	0.028	
Mode of delivery		–3.28 (–34.59 to 28.02)	0.832	
Residual cortical gray matter volume (mL)	0.00 ± 13.4			0.13
IGF‐1 (nmol/L)		1.46 (0.31 to 2.61)	0.015	
Mode of delivery		–0.24 (–15.83 to 15.35)	0.975	
Residual cerebellar volume (mL)	0.00 ± 2.08			0.07
IGF‐1 (nmol/L)		0.19 (0.00 to 0.37)	0.051	
Mode of delivery		–0.97 (–3.48 to 1.54)	0.435	
Residual deep gray matter volume (mL)	0.00 ± 1.71			0.15
IGF‐1 (nmol/L)		0.20 (0.05 to 0.34)	0.009	
Mode of delivery		–0.23 (–2.19 to 1.73)	0.814	
Residual white matter volume (mL)	0.00 ± 11.20			0.00
IGF‐1 (nmol/L)		0.72 (–0.32 to 1.75)	0.166	
Mode of delivery		–1.72 (–15.71 to 12.26)	0.803	
IGFBP‐3 and brain volume				
Residual total brain volume (mL)	0.00 ± 26.36			0.06
IGFBP‐3 (mg/L)		45.30 (–0.17 to 90.78)	0.051	
Mode of delivery		0.43 (–31.14 to 32.01)	0.978	
Residual cortical gray matter volume (mL)	0.00 ± 13.4			0.07
IGFBP‐3 (mg/L)		23.46 (0.44 to 46.47)	0.046	
Mode of delivery		1.88 (–14.10 to 17.86)	0.812	
Residual cerebellar volume (mL)	0.00 ± 2.08			0.11
IGFBP‐3 (mg/L)		4.12 (0.62 to 7.62)	0.023	
Mode of delivery		–0.73 (–3.16 to 1.70)	0.542	
Residual deep gray matter volume (mL)	0.00 ± 1.71			0.01
IGFBP‐3 (mg/L)		2.23 (–0.80 to 5.25)	0.144	
Mode of delivery		0.08 (–2.02 to 2.19)	0.935	
Residual white matter volume (mL)	0.00 ± 11.20			0.01
IGFBP‐3 (mg/L)		14.59 (–5.30 to 34.47)	0.145	
Mode of delivery		–0.77 (–14.57 to 13.04)	0.911	

Residual brain volume indicates the difference between the measured brain volume of each case and their expected value, calculated from linear regression models that include absolute brain volume (dependent variable), neonatal sex (independent variable) and postmenstrual age at magnetic resonance imaging (independent variable). Reference category for mode of delivery was vaginal delivery in these analyses. *P* < 0.05 indicates statistical significance.

### Perinatal brain growth and IGF‐1/IGFBP‐3 levels

Fetal MRI was performed in 32 (82%) CCHD cases, of which 26 scans met the criteria for sufficient quality of both the reconstruction and segmentation of fetal brain tissues (Figure 1). The median postmenstrual age at scanning for these cases was 32.9 (IQR, 32.0–34.2) weeks.

Longitudinal MRI data were available in 22/39 (56%) cases (lower COD, *n* = 19; normal COD, *n* = 3), with a median time interval of 6.5 (IQR, 5.0–7.0) weeks between the fetal and neonatal MRI scans. IGF‐1 demonstrated a positive correlation with fetal‐to‐neonatal cortical gray matter growth per week (*r* = 0.47; *P* = 0.039). No correlations were observed between IGFBP‐3 and volumetric growth rates per week.

## Discussion

In this exploratory study, we prospectively examined cord blood levels of IGF‐1 and IGFBP‐3 at birth in neonates with CCHD and healthy controls. Our results showed that IGFBP‐3 is lower in neonates with CCHD. When stratifying cases based on their predicted COD *in utero*, both IGF‐1 and IGFBP‐3 levels were lower in cases with lower COD. Using high‐quality fetal and postnatal MRI data, we explored the relationship between IGF‐1 and IGFBP‐3 levels and early brain development in neonates with CCHD. Positive associations were observed between IGF‐1 and IGFBP‐3 levels and postnatal cortical gray matter, deep gray matter, cerebellar and total brain volumes, with IGF‐1 also showing a positive correlation with fetal‐to‐neonatal cortical gray matter growth.

### IGF‐1/IGFBP‐3 and early brain development

To our knowledge, this is the first study evaluating the association between IGF‐1 and IGFBP‐3 levels and perinatal brain development in cases of CCHD. We have shown that lower IGF‐1 and IGFBP‐3 levels are associated with reduced regional and total brain volumes in the CCHD population. IGF‐1 is an essential regulator of fetal brain development, facilitating processes such as proliferation, differentiation and maturation of neural progenitor cells, while also exerting an antiapoptotic effect^14^, ^15^. IGFBP‐3 not only stimulates IGF‐1 activity, but also has independent neurotrophic effects on cell differentiation and survival^16^. Our results add to the existing literature on preterm infants that shows associations between reduced IGF‐1 and IGFBP‐3 levels and smaller regional and total brain volumes^26^, ^27^. Additionally, decreased IGF‐1 levels have been shown to be correlated with poorer neurodevelopmental outcomes at 2 years of age in preterm infants^28^. IGF‐1 and IGFBP‐3 levels are known to peak in the third trimester, supporting the accelerated fetal growth in this developmental period^13^. Interestingly, we observed a positive association between IGF‐1 and IGFBP‐3 levels and gray matter, cerebellum and total brain volumes, but no association for either factor was observed with white matter volume. This may imply that the brain regions which undergo the most pronounced volumetric growth throughout the third trimester may be more vulnerable for disruption in the IGF axis^39^.

### The IGF axis and hypoxia

In our cohort, decreased IGF‐1 and IGFBP‐3 levels were detected in the umbilical vein in cases with lower predicted COD. Previous studies have highlighted that cases with lower COD are more susceptible to compromised early brain development^4^, ^29^, ^30^. Because of the limited sample size of our study, it is challenging to determine if the linear association we observed between IGF‐1 and IGFBP‐3 levels and perinatal brain volumes reflects the inherent relationship between lower COD and smaller brain volume or if reductions in the IGF axis actively contribute to delayed brain development in this group.

Hypoxia initiates a cascade of events via hypoxia‐inducible factor mediated pathways, which result in upregulation of insulin‐like growth factor binding protein‐1 (IGFBP‐1) expression^40^, ^41^. While IGFBP‐3 stimulates IGF‐1 distribution and bioavailability, IGFBP‐1 predominantly inhibits IGF‐1 activity, thereby negatively affecting growth^16^, ^41^, ^42^. Previous clinical studies have demonstrated these negative effects of hypoxia on the IGF system. For instance, in preterm infants, umbilical artery partial pressure of oxygen (pO_2_) levels were reported to be associated positively with umbilical vein IGF‐1 concentrations and inversely with IGFBP‐1 concentrations^18^. Similarly, in children with acute respiratory distress, IGF‐1 and IGFBP‐3 levels were found to be decreased, while IGFBP‐1 levels were increased^17^. In older pediatric patients with CHD, IGF‐1 and IGFBP‐3 were found to be lower compared to healthy controls^21^, ^22^, ^23^, ^24^, ^25^, with some studies specifically noting positive associations between these factors and the oxygenation profile^21^, ^22^.

### Future perspectives

Our findings imply that umbilical vein IGF‐1 and IGFBP‐3 may be valuable as early biomarkers for adverse neurodevelopment in neonates with CCHD. In addition, targeting the IGF axis may be a promising approach in supporting early brain development in CCHD. In several *in‐vivo* studies, IGF‐1 administration following hypoxic‐ischemic events prevented brain injury, including loss of neuronal and oligodendrocyte progenitor cells, and reduced neurobehavioral deficits^43^, ^44^, ^45^. In a phase‐2 trial involving extremely preterm infants (delivered < 28 weeks), systemic IGF‐1/IGFBP‐3 therapy led to a decrease in the occurrence of severe bronchopulmonary dysplasia and a non‐significant decrease in moderate‐to‐severe intraventricular hemorrhage^46^. The effects of IGF‐1/IGFBP‐3 therapy on neurodevelopmental outcomes in this population are currently being investigated (NCT03253263). As for maternal administration, since maternal IGF‐1 does not cross the placental barrier, its anticipated influence on fetal growth is mediated through maternal and placental adaptations^47^. Experimental studies have indicated that maternal IGF‐1 therapy enhances placental nutrient transport and fetal growth^48^, ^49^, ^50^, but the effects on fetal brain development have not yet been explored.

### IGF‐1/IGFBP‐3 and somatic growth

Aside from brain development, the IGF axis also plays a critical role in somatic growth during fetal development^13^. In our study, IGF‐1 was associated with birth‐weight *Z*‐score, reaffirming the findings from previous research on this topic in pediatric CHD cases^23^, ^24^, ^25^. However, it is important to consider the potential bidirectional nature of this association. While diminished IGF‐1 levels can lead to poor growth of the fetus, fetal weight itself may also influence IGF‐1 synthesis. Interestingly, we did not observe a significant relationship between IGFBP‐3 and birth‐weight *Z*‐score. It can be speculated that IGFBP‐3 synthesis may be less reliant on fetal weight, potentially making it a more sensitive marker for assessing the effects of hypoxia on the IGF‐1/IGFBP‐3 axis in CCHD.

### Limitations

There are a number of limitations to consider in this study. First, the sample size may hinder the generalizability of our findings and it limited our ability to explore the association of IGF‐1 and IGFBP‐3 levels with brain volumes within each COD subgroup, because we were unable to perform separate linear regression analyses for each subgroup; it also limited our ability to fully account for confounding factors. Second, as all neonates in the control cohort were delivered via elective Cesarean section, there was a disparity in birth mode between the control and CCHD cohorts. Because of this study design, birth mode in our population inherently reflects cohort assignment; therefore, we could not adjust the comparative analyses between the control and CCHD cohorts for birth mode. However, we did account for birth mode in the analyses that examined the association of IGF‐1 and IGFBP‐3 levels with brain volume. While some studies have indicated that birth mode can influence umbilical vein levels of IGF‐1^51^, ^52^, other studies did not find this association^53^, ^54^. With regard to IGFBP‐3 levels and birth mode, no association has been observed^52^, ^53^, suggesting that IGFBP‐3 is possibly a more robust marker for assessing the impact of hypoxia on the IGF axis. Third, we measured IGF‐1 and IGFBP‐3 levels at a single timepoint; since the levels of these factors typically rise over the course of gestation^13^, the IGF‐1 and IGFBP‐3 levels recorded directly after birth do not fully reflect the IGF axis throughout the intrauterine period. Fourth, aside from *in‐utero* oxygenation, the IGF axis is also influenced by maternal health and placental function, both of which were not accounted for in this study due to the small sample size. Fifth, the cord blood samples we obtained comprised circulating IGF‐1 and IGFBP‐3 levels in the fetus instead of brain‐derived levels. However, it is worth noting that systemic IGF‐1 and IGFBP‐3 do cross the blood–brain barrier and their significance to central nervous system function has been recognized^55^. Sixth, a limitation of the fetal‐to‐neonatal growth rate analysis is that averaging growth over the fetal–neonatal MRI interval may have resulted in non‐linear growth patterns being overlooked. Lastly, we classified cases according to their predicted fetal COD rather than measuring COD quantitatively. In the future, relating fetal MRI oximetry to the IGF axis may offer valuable insights into the interplay between the fetal oxygenation profile and IGF‐1/IGFBP‐3 regulation.

### Conclusions

IGFBP‐3 levels were reduced in neonates with CCHD, and cases with lower predicted COD demonstrated decreased levels of both IGF‐1 and IGFBP‐3. In addition, this study found that lower IGF‐1 and IGFBP‐3 levels at birth are associated with smaller regional brain volumes. Collectively, our findings suggest that the compromised *in‐utero* oxygenation profile of fetuses with CCHD may affect the IGF axis, possibly contributing to the impaired brain growth observed in this population. Larger follow‐up studies are essential to more effectively address the confounding factors and limitations of this pilot study.

## Data Availability

The data that support the findings of this study are available from the corresponding author upon reasonable request.
